# Characteristics of particle-bound polycyclic aromatic hydrocarbons (PAHs) in indoor PM_2.5_ of households in the Southwest part of Ulaanbaatar capital, Mongolia

**DOI:** 10.1007/s10661-022-10297-0

**Published:** 2022-08-11

**Authors:** Tsend-Ayush Sainnokhoi, Nora Kováts, András Gelencsér, Katalin Hubai, Gábor Teke, Bolormaa Pelden, Tsagaan Tserenchimed, Zoljargal Erdenechimeg, Jargalsaikhan Galsuren

**Affiliations:** 1 Centre for Natural Sciences, University of Pannonia, Egyetem street 10, Veszprém, 8200 Hungary; 2 Research Institute of Biomolecular and Chemical Engineering, University of Pannonia, Egyetem str. 10, Veszprém, 8200 Hungary; 3 ELGOSCAR-2000 Environmental Technology and Water Management Ltd, 8184 Balatonfűzfő, Hungary; 4 School of Veterinary Medicine, Mongolian University of Life Sciences, Khan-Uul District, 17042 Ulaanbaatar, Mongolia; 5 School of Public Health, Mongolian National University of Medical Sciences, Zorig street, Ulaanbaatar, 14210 Mongolia

**Keywords:** Ulaanbaatar, Indoor air quality, PM_2.5_, Polycyclic aromatic hydrocarbons, Cancer risk, Ecotoxicity, Bioassay

## Abstract

Air pollution, including PM_2.5_ concentration in Ulaanbaatar (capital of Mongolia) is a serious matter of concern. As the majority of households use coal in large areas of the city, indoor air quality is also posing a serious risk to human health. This study investigated the concentration of polycyclic aromatic compounds (PAHs) in indoor particulate matter (PM_2.5_) in 10 non-smoker households. Sampling was conducted in winter of 2018, between 27 January and 09 February. Concentrations of PM_2.5_ in the indoor air of households ranged between 62.8 and 324.8 µg m^−3^. Total concentration of PAHs also varied in a relatively wide range, between 46.2 and 175.7 ng m^−3^. Five-ring PAHs represented a considerably high fraction of total PAHs between 25 and 53%, benzo[b]fluoranthene (BbF) and benzo[a]pyrene (BaP) were the two predominant compounds within five-ring PAHs. Significant correlation was found between indoor and outdoor particulate matter levels in wintertime. Considering individual characteristic PAHs, heavier PAHs homologues (4- to 5-ring and 6-ring PAHs) were detected in all households, which suggested the influence of coal combustion and traffic exhaust. Health risk of children attributed to PAHs inhalation was assessed by taking into account the lifetime-average daily dose (LADD) and corresponding lifetime cancer risk. Lifetime average daily dose for children in only one household were slightly higher than health-based guideline level (1.0 × 10^−5^), defined by WHO, whereas LADD for adults and children of other households were within acceptable limit. The cancer risks from the exposure of children to air pollutants in all households except HH-3 were found high. In the *Vibrio fischeri* bioluminescence inhibition assay, according to the toxic unit (TU) values of indoor PM_2.5_ from ten households, all samples were classified as toxic.

## Introduction

Air quality in Ulaanbaatar (capital of Mongolia) is a serious matter of concern. Threshold levels are established for 10-μm-diameter (PM_10_) and 2.5-μm-diameter (PM_2.5_) particles. Since 2009, PM_2.5_ has been recorded to be higher than the standard of WHO (WHO, 2021) and Mongolian National Ambient Air Quality Standard (MNS 4585/2007, Mongolian Agency for Standardization and Measurement, 2008) which prescribes 50.0 µg m^−3^ 24-h ambient PM_2.5_ concentration and 25.0 μg m^−3^ 1-year ambient PM_2.5_ concentration (Batmunkh et al., 2013). The World Air Quality report listed Ulaanbaatar as having worst air quality in the world (WIAQ, 2020).

According to a World Bank survey (World Bank, 2013), 98% of households use coal in ger areas of Ulaanbaatar (‘ger’ refers to traditional Mongolian yurt; more than 60% of the population of Ulaanbaatar lives in gers in peripheral area called ger area or ger district without primary service). Unprocessed coal is often burned inside poorly ventilated spaces with traditional stoves. Combustion of solid fuels is the main source of elevated levels of PM and polycyclic aromatic hydrocarbons (PAHs) in indoor environments. In a South African study, in winter 24-h average PM_4_ concentration in a solid fuel burning (SFB) house was about 3–4 times higher than in a non-solid fuel burning (NSFB) house (Adesina et al., 2020). Chinese studies also report that on the average, concentration of fine particles (PM_2.5_ and PM_4_) is app. twice in SFB residential homes (Du et al., 2018). Twenty-four-hour personal exposure to PM_2.5_ was measured in a study of Secrest et al. (2016) involving rural women in Inner Mongolia who typically used biomass fuels for cooking and lignite for heating. The geometric mean PM_2.5_ exposure was as high as 249 µg m^−3^.

According to Allen et al. (2013), up to 25% of deaths are caused by PM pollution annually in Ulaanbaatar. Respiratory diseases have been widely reported such as asthma (Po et al., 2011), lung inflammation (Wang et al., 2015), decreased lung immunity (Feng et al., 2016), and resulting bacterial infections (Zhao et al., 2014). Chronic obstructive pulmonary disease (COPD) and lung cancer were associated with women who were exposed during cooking with solid fuels (Barabad et al., 2018). In 2015, more than 430 children under the age of 5 years died in pneumonia in Ulaanbaatar. Children living in a highly polluted district of Ulaanbaatar were found to have app. 40% higher incidence of lung diseases than children living in a rural area (UNICEF, 2018).

In addition to respiratory problems, winter ambient air pollution was found to correlate strongly with spontaneous abortion in a Mongolian study (Enkhmaa et al., 2014) as well as reduced fecundity (Badarch et al., 2021). A meta-analysis revealed that the use of household solid fuel can be significantly associated with an increased risk of hypertension (Li et al., 2020). Neurodevelopmental disorders in Mongolian children have also been reported (Jadambaa et al., 2015).

Indoor PM_2.5_ levels should deserve more attention, following some indoor pollutant studies such as coal combustion in yurt district during winter time (Lim et al., 2018), determination of indoor PM_2.5_ concentration in Mongolian traditional yurt (Ahn et al., 2019; Ban et al., 2017), as well as characteristics of lifestyles and living condition (So et al., 2019). Lim et al. (2018) reported that the 24-h average PM_2.5_ concentration was 203 µg m^−3^ in yurt with conventional stove, whereas 257 µg m^−3^ in yurt with improved stove. On the other hand, it is possible that high levels of ambient air pollution have a negative impact on indoor air quality, including PM_2.5_ concentration (e.g., Bai et al., 2020; Hu et al., 2018; So et al., 2019; Sonomdagva et al., 2017).

Airborne particles bind potentially toxic compounds such as PAHs which are generated by incomplete combustion of fossil fuels, in urban and rural environments the major sources are household heating and transportation (Gelencsér et al., 2007; Nagy & Szabó, 2019; Pandey et al., 2013). An early study revealed that the concentration of PAHs in indoor air samples highly varied with the aerodynamic diameter of the particles, fine particles contained high concentrations of PAH and mutagens (Ando et al., 1996). In a study conducted in Athens during the winter and summer periods of 2003–2004, Valavanidis et al. (2006) also demonstrated that concentration of particulate-bound PAHs was higher in fine particles than in coarse particular matter. Hassanvand et al. (2015) investigated PAH concentrations and profiles in indoor PM_10_, PM_2.5_, and PM_1_ in a retirement home and a school dormitory in Tehran and reported that the total PM-bound PAHs were predominantly found in the PM_2.5_ fraction.

The US Environmental Protection Agency (EPA) registered 16 priority PAHs that were identified as probable human carcinogens and posing the highest environmental risk (reviewed by Abdel-Shafy & Mansour, 2016). Some PAHs, such as chrysene, benzo[a]anthracene, and benzo[a]pyrene are procarcinogens. Ohura et al. (2005) reported that these carcinogenic PAHs are associated with PM_2.5_.

People spend on an average as much as 87% of their time in enclosed buildings (Klepeis et al., 2001), which makes public health issue in the built environment a top priority (Sojobi & Liew, 2022; Sojobi & Zayed, 2022). They are often ignorant that they can be constantly exposed to air pollution, especially in cold regions (Leech et al., 2002). As Mongolia is located at high latitude, having dry and cold continental climate, people spend quite little time outside in winter as they prefer warm places, similarly to inhabitants of other cold countries. Naturally, during this time, fuel usage increases significantly.

In order to characterize the ecotoxicity of particle-bound potentially toxic compounds, the bioassay based on the bioluminescence inhibition of the marine bacterium *Vibrio fischeri* has been widely used (reviewed by Kováts & Horváth, 2016). Most studies, however, discuss only outdoor pollution, much less works have addressed indoor ecotoxicity (e.g. Alves et al., 2021).

**Fig. 1:**
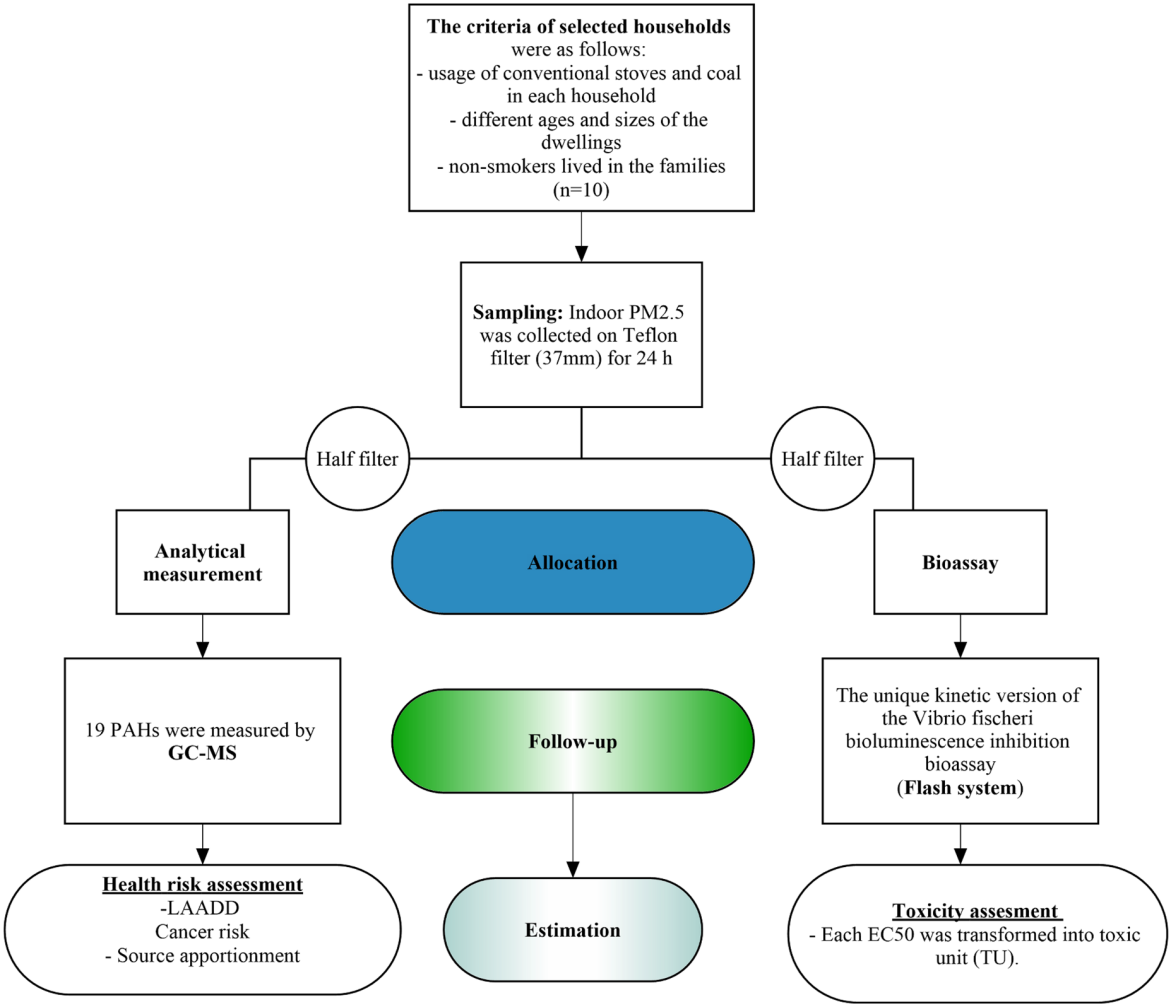
Flowchart of the study

Taking into consideration that practically no data exist on health risk of indoor air pollution from Mongolia, our study was targeted to assess indoor air quality (PM_2.5_ concentration, PAH distribution pattern, and resulting ecotoxicity as well as cancer risk estimation) in 10 households located in the region around the Khan-Uul (one of the districts in Ulaanbaatar capital). Contrary to exceptionally polluted districts, the sample area can be regarded as average regarding ambient air quality (Sonomdagva et al., 2017). Figure 1 shows the flowchart of the steps completed within the study.

## Materials and methods

### Sampling site

**Fig. 2:**
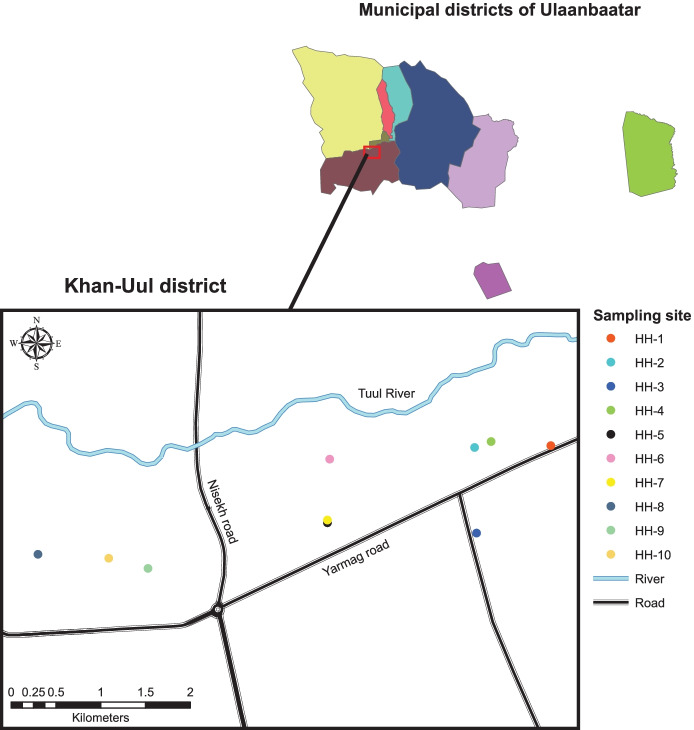
Map showing the sampling area within Khan-Uul district, Ulaanbaatar, Mongolia

The criteria of selected households for our study were as follows: 60% of Mongolians live in the ger area (Guttikunda et al., 2013), app. half of the dwellings in the ger area are made of brick or clay, typically constructed by the owner of each house. We wanted to represent both types, as well as different size of households and different ages of the dwellings. On the other hand, similarities were location relatively close to the main road; heating type (usage of conventional stoves and coal in each household); and only non-smokers lived in the selected families. Naturally, consent of the families was also an important factor. Ten households were selected in Khan-Uul district, Ulaanbaatar (hereinafter named HH-1 to HH-10). Households (HH-8, HH-9, and HH-10) were located app. 500 m from main road, whereas HH-2, HH-4, HH-5, and HH-7 were app. 200 m. Other three households were located between 50 m and 1 km from main road (HH-1—50 m, HH-3—300 m, and HH-6—1 km). A map of the sampling locations is shown in Fig. 2.

In addition, basic characteristics of sample sites (households) are given in Table 1 which were used to identify the factors that might affect indoor air quality.

**Table 1:** Description of sampling sites

	**Concentration of indoor PM2.5 (µg/m**^**3**^**)**	**House type**	**Residency year**	**Sampling place**	**House-holds area (m**^**2**^**/room number)**	**Family members**	**Heater type**	**Ventilation per day**	**Heating time per day**	**Cooking per day**	**Nearby outdoor PM source**	**Tobacco smoke**
HH-1	107.2	Fired clay brick	12	Living room	60/3 rooms	5	Stove	–	Coal, processed coal and wood/4	3	Next to main road	No
HH-2	320.7	Breeze-block	12	Living room	27/2 rooms	7	Stove	1	Coal, processed coal and wood/3	1	Main road, unpaved road	No
HH-3	176.5	Fired clay brick	7	Living room	64/3 rooms	5	Electric/stove	1	Coal, processed coal and wood/6	2	Next to unpaved road	No
HH-4	137.2	Breeze-block	10	Sleeping room	71/2 rooms	8	Stove	2	Coal, processed coal and wood/2	3	Next to unpaved road	No
HH-5	93.6	Fired clay brick	4.5	Living room	31.15/1 room	5	Stove	–	Coal, processed coal and wood/3–4	3	Next to mini power plant, main road	No
HH-6	324.8	Fired clay brick	45	Living room	160/2 rooms	7	Electric/ stove	–	Coal, processed coal and wood/-	2	Unpaved road	No
HH-7	214.6	Clay and breeze-block	40	Living room	24/1 room	6	Stove	–	Coal, processed coal and wood/3	1	Next to mini power plant, main road	No
HH-8	246.2	Fired clay brick	10	Living room	18/1 room	6	Stove	–	Coal, processed coal and wood/4	-	Unpaved road	No
HH-9	62.8	Breeze-block	26	Living room	104/3 rooms	9	Electric/ stove	–	Coal, processed coal and wood/3	2	Next to unpaved road	No
HH-10	142.8	Breeze-block	50	Kitchen room	32/1 room	3	Electric/ stove	–	Coal, processed coal and wood/2	2	Next to unpaved road	No

Fine indoor particulate matter (PM_2.5_) samples were collected on Teflon filter between 27 January and 09 February 2018 using AirChek XR5000 (SKC Ltd.). The flow rate for sampling was 1.5–2.0 L min^−1^ for 24 h, the instrument was placed at 1 to 1.5 m above ground level to simulate breathing zone.

Indoor PM_2.5_ samples were stored in labeled plastic cassette in a cooler box filled with dry ice and transported to the laboratory at the Department of Public Health, Mongolian National University of Medical Sciences. After gravimetric measurement of PM_2.5_ mass, samples were kept in the freezer at – 20 °C before being transported for further processing to the Centre of Natural Sciences, University of Pannonia, Hungary.

### Analytical measurements

Polycyclic aromatic hydrocarbon (PAH) concentrations were measured by gas chromatographic mass spectrometry (Agilent 6890GC 5973E MSD GC–MS according to MSZ (Hungarian Standard) 1484–6:2003). One half of the filters was extracted with 20 ml *n*-hexane three times for 20 min in a sonication bath. Prior to extraction, 10 ml acetone was added and the samples were spiked with 100 µl of 0.01 µg ml^−1^ deuterated PAHs surrogate mixture containing Naphtalene-d_8_, Acenaphtalene-d_10_, Phenanthrene-d_10_, Chrysene-d_12_, Benzo(a)pyrene-d_12_, and Perylene-d_12_ (Resteck Corporation, USA). Extracts were concentrated in a dry nitrogen stream to a volume of 1 ml; the clean-up of each sample was completed via alumina oxide and solid phase silica gel. For GC–MS measurements, an HP-6890 gas chromatograph was coupled to an HP-5973 quadrupled mass spectrometer (low-resolution single MS) (Agilent Technologies, Palo Alto, USA). The concentration of 19 individual PAHs including US EPA priority pollutants (Naphthalene (Nap), 2-methyl Naphthalene (Methy-Nap), 1-methyl Naphthalene (Me-Nap), Acenaphthylene (Acl), Acenaphthene (Ace), Fluorene (Fle), Phenanthrene (Phe), Anthracene (Ant), Fluoranthene (Flu), Pyrene (Pyr), Benz[a]anthracene (BaA), Chrysene (Cry), Benzo[b]fluoranthene (BbF), Benzo[k]fluoranthene (BkF), Benzo[e]pyrene (BeP), Benzo[a]pyrene (BaP), Dibenzo[a,h]anthracene (DBahA), Indeno[1,2,3CD]pyrene (IDP), Benzo[g,h,i]perylene (BghiP)) were analyzed in each household indoor air sample.

For quality assurance/quality control, (QA/QC) internal standard (p-Terphenyl-d14, 2-fluorobiphenyl from Restek Corporation, Bellefonte, Pennsylvania US) and surrogate standard (Naphtalene-d8, Acenaphthene-d10, Phenanthrene-d10, Chryzene-d12 Benzo(a)pyrene-d12, and Perylene-d12, from Restek Corporation, Bellefonte, Pennsylvania US) were used for quantification and quantifying of sample and for procedural recovery. Before the analysis, standards were freshly prepared and diluted with GC grade solvents (Sigma-Aldrich, St. Louis, Missouri USA). Recoveries for the compounds ranging between 73.5 and 119.4%, this achieved good by the regulatory requirements of the USA-EPA and EU. In our measurement, the recoveries were 96–104% for 2-fluorobiphenyl and 108–114% for p-Terphenyl-d14. The recoveries of surrogate standards were acceptable for the standards (Naphtalene-d8, Acenaphthene-d10 82–102%, Phenanthrene-d10 92–109%, Chrysene-d12 95–107%, perylene-D12 82–91%), which were good for making results reliable.

Analytical determinations were performed by courtesy of the Laboratory of the ELGOSCAR-2000 Environmental Technology and Water Management Ltd. accredited by the National Accreditation Authority (complies with criteria of Standard MSZ EN ISO/IEC 17,025:2018), registration number NAH-1–1278/2015.

### Health risk characterization

Health risk assessment can be established using PAHs exposure, based on one of the approaches is inhalation exposure. In our case, inhalation of indoor air particles containing PAHs was assessed via the use of toxicity equivalency factor (TEFs) based on BaP, and estimated BaP equivalent concentration (BaPeq) (Bari et al., 2010). The list of TEFs composed by Lu et al. (2008) was adopted (Fig. 6), and the total PAH-associated carcinogenicity was calculated based on the following formula:

BaP_eq_ = ∑ (C_i_ × TEF) where C_i_ is concentration of PAHs in indoor PM_2.5_ sample.

Lifetime average daily dose (LADD) of PAHs was calculated based on the guideline of Boström et al. (2002) as follows:

$$\mathrm{LADD }(\mathrm{mg}/\mathrm{kg}/\mathrm{day})=\frac{Ci\ x\ IR\ x\ ET\ x\ EF\ x\ ED}{BW\ x\ AT}\times \mathrm{CF}$$

Cancer risk was estimated as follows: incremental lifetime cancer risk (ILCR) = Lifetime average daily dose (LADD) × cancer slope factor (CSF).

LADD is intake of chemical compounds suspected for causing adverse health effects expressed as mg kg^−1^ of bodyweight per day. In general, chronic exposure is assumed. C_*i*_ is concentration of particular PAHs (ng m^−3^); IR is the intake rate (for an adult 0.83 m^3^ h^−1^; for up to 14 year child—0.87 m^3^ h^−1^); ET is exposure time (21 h day^−1^); EF is the exposure frequency (350 days year^−1^). ED represents exposure duration, 70 years for adults and 14 years for children. CF is the unit of the conversion factor (10^−6^). BW is the average body weight (for adults: – 70 kg, for children: 59.4 kg), AT represents the average timing (for adults: 25,550 days (70 × 365); for children:– 5110 days (14 × 365)) (Bozek et al., 2009; Iwegbue et al., 2019; Ortega-García et al., 2017). In this case, we used values of cancer slope factor for carcinogenic-PAHs, which were compiled (USEPA, 1992), and the LADD and ILCR estimations for adult and child in indoor air sample were also presented in Table 3.

### Source apportionment

Calculation of certain PAHs diagnostic ratio is widely used technique to estimate the presented PAHs origin in various environment media such as air sample. Diagnostic ratios were calculated as follows: fluoranthene to fluoranthene plus pyrene [Flt/(Flt + Pyr)]; benz[a]anthracene to benz[a]anthracene plus chrysene [BaA/(BaA + Cry)]; indene[1,2,3-cd]pyrene to indene[1,2,3-cd]pyrene plus benzo[ghi]perylene [Ind/(Ind + BghiP)] (Yunker et al., 2002).

### Toxicity assessment

Ecotoxicity testing was carried out according to ISO 21338:2010: water quality – kinetic determination of the inhibitory effects of sediment, other solids and colored samples on the light emission of *Vibrio fischeri* (kinetic luminescent bacteria test). The kinetic protocol was specially designed to measure the light output of test bacteria in turbid or colored samples. Prior to measurement, freeze-dried inactivated luminous *V*. *fischeri* (NRRL-B-11177, supplier Hach Lange Co.) were rehydrated with reconstitution solution (glucose/sodium chloride, buffered to PH 7.0 in a PE bottle) and then incubated at 15 °C for 40 min.

Measurements were done in 96 multi-well plate using 1:2 dilutions in 2 replicates. After the sample was injected into the bacterial suspension, bioluminescence intensity was continuously recorded for the first 30 s. After 30 min of exposure, luminescence intensity was read again. The light output of the unstressed bacteria (the first 30 s) was used as a reference in calculating the results. EC_50_ (calculated percent concentration of original extract which causes 50% of effect) values were calculated from the light inhibition percentages by the Aboatox software provided with the Luminoskan Ascent Luminometer (Thermo Scientific).

Each EC_50_ was transformed into dimensionless toxic unit (TU) calculated using the formula as follows (Chang et al., 2013):

TU = (1/EC_50_) × 100%

TU categories are generally classified as: non-toxic (< 1), toxic (1–10), very toxic (10–100), extremely toxic (> 100), respectively.

## Results and discussion

### Concentration of PM_2.5_ in indoor air of households

Concentrations of PM_2.5_ ranged between 62.8 and 324.8 µg m^−3^. The highest concentrations were found in HH-6 (324.8 µg m^−3^), HH-2 (320 µg m^−3^), and HH-8 (246.2 µg m^−3^), while the lowest concentrations in HH-5 (93.6 µg m^−3^) and HH-9 (62.8 µg m^−3^) (see Table 1). However, values of all household indoor air significantly exceeded limits of WHO (10 µg m^−3^). Kim et al. (2021) compared personal PM_2.5_ exposure of Ger residents vs. apartment residents and found that the first group was exposed to a significantly higher PM_2.5_ concentration.

### PAH concentrations in indoor air of households

Concentrations of 19 individual PAHs in indoor air of each household were measured (Table 2); the highest total concentrations were found in HH-7 (175.7 ng m^−3^), followed by HH-2 (137.3 ng m^−3^), HH-5 (105.1 ng m^−3^), HH-4 (74.1 ng m^−3^), HH-1 (70.2 ng m^−3^), HH-10 (69.7 ng m^−3^), HH-6 (69.5 ng m^−3^), HH-8 (63.0 ng m^−3^), and HH-3 (46.7 ng m^−3^). The lowest concentration (46.2 ng m^−3^) was detected in HH-9.

**Table 2:** Concentration (ng m^−3^) of PAHs in the indoor PM_2.5_ samples of households, carcinogenic PAHs are given in italic. (ND: not detected)

	**HH-1**	**HH-2**	**HH-3**	**HH-4**	**HH-5**	**HH-6**	**HH-7**	**HH-8**	**HH-9**	**HH-10**
Naphthalene	6.2	5.4	7.0	6.1	6.2	5.1	4.7	5	5.8	5.6
2-methyl-naphthalene	5.6	4.5	6.2	5.8	6.2	5.4	4.6	4.7	6.5	5.3
1-methyl-naphthalene	5.5	1.9	2.4	2.5	6.1	2.2	1.9	1.85	2.7	2.3
Acenaphthylene	0.4	0.5	0.3	0.4	ND	0.4	0.6	0.25	0.3	0.7
Acenaphthene	0.2	0.1	0.6	0.3	ND	0.2	0.2	0.15	0.2	0.2
Fluorene	1.1	0.8	1.4	0.9	0.8	0.8	0.8	0.85	0.9	0.8
Phenanthrene	6.7	6.5	7.0	7.9	5.4	5.5	6.1	5.5	4.4	5.3
Anthracene	0.2	0.4	0.2	0.3	7.3	0.3	0.4	0.4	0.2	0.3
Fluoranthene	2.3	5.3	2.4	5.2	6.3	0.3	7.4	3	1.4	2.0
Pyrene	1.6	4.4	1.4	2.4	10.1	2.5	8.1	2.95	1.3	1.7
*Benzo (a)anthracene*	2.1	9.0	1.1	2.3	6.9	4.3	13.7	3.95	1.5	2.8
*Chrysene*	1.5	7.1	0.9	5.5	5.9	3.3	9.0	3.25	1.2	2.4
*Benzo(b)fluoranthene*	11.5	28.8	4.5	12.9	13.1	13.1	37.7	11.4	5.6	11.8
*Benzo(k)fluoranthene*	3.4	8.9	1.0	2.4	3.8	3.9	12.6	2.85	2.2	3.6
*Benzo(e)pyrene*	5.3	12.2	1.8	3.4	7.7	5.9	17.5	5.35	2.7	6.6
*Benzo(a)pyrene*	7.8	20.4	4.1	6.9	9.5	8.3	25.1	5.3	4.8	8.9
*Dibenzo[a.h] anthracene*	1.1	1.6	0.4	2.1	ND	0.5	1.7	0.75	0.3	0.8
*Indeno1.2.3CD-Pyrene*	4.4	11.8	2.4	4.0	5.4	4.9	14.4	2.95	2.6	5.1
*Benzo(g.h.i)perylene*	3.0	7.7	1.7	3.4	4.8	3.0	9.2	2.6	1.6	3.5
Total PAH	70.2	137.3	46.7	74.1	105.1	69.5	175.7	63	46.2	69.7

Although PAH concentrations in winter samples collected in warm regions might also reach rather high values (e.g., Li and Ro, 2000), for our study comparison with colder regions seems to be more relevant, as the contribution from home heating and more representative meteorological conditions can be taken into consideration. Mohammed et al. (2016) investigated the distribution patterns of PM_2.5_-bound PAHs in indoor samples collected in Harbin city (northeastern China). The mean concentration of the sum of 16 US EPA priority PAHs was 102 ± 75 ng m^−3^. The concentration of total PAHs in the PM_2.5_ fraction of indoor school samples collected in a campaign during the heating season in Kaunas (Lithuania) ranged from 20.3 to 131.1 ng m^−3^ (Krugly et al., 2014). In a comparative study, Lu et al. (2011) measured total concentration of 8 PAHs which amounted to 320 ng m^−3^ in residential air of Hangzhou (China). In an Indian study, the average PAH concentration in residential homes was 233 ng m^−3^ in winter (Masih et al., 2012). Li et al. (2017) measured an average of 39.6 ng m^–3^ PAH concentration in indoor PM_2.5_ samples collected in January from suburban hotels in Jinan (China).

While our results fall into a similar range, some studies report exceptionally high concentrations: e.g., Zhu et al. (2009) measured as high as 36.200 ng m^−3^ total concentration of PAHs in residential air in Hangzhou (China).

**Fig. 3:**
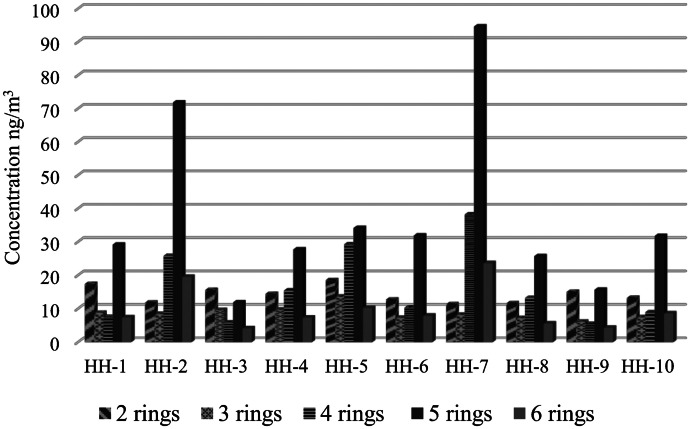
Total amount of different molecular weight PAHs in households

Figure 3 shows the total amount of different molecular weight PAHs in each household. Concentrations of heavy PAHs (five- and six-rings) were significantly higher in HH-7 and HH-2 than in others. It should be noted that prevalent group was five-ring PAHs in indoor air of all households except HH-3. The results indicated that benzo[b]fluoranthene (BbF) and benzo[a]pyrene (BaP) represented a significant fraction of five-ring PAHs in each sample.

**Fig. 4:**
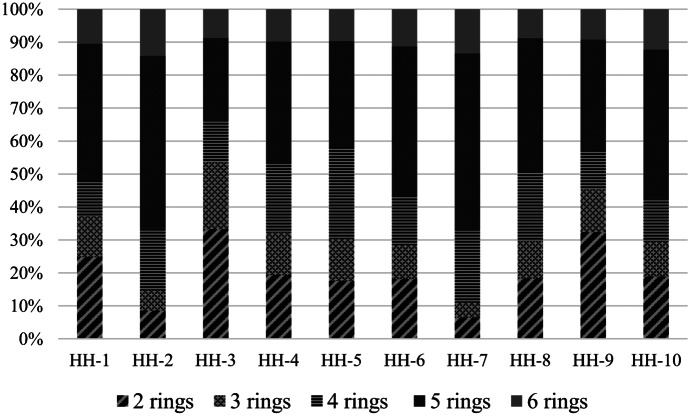
Percentage contribution of different molecular weight PAHs in the households

The percentage contribution of different molecular weight PAHs is shown in Fig. 4. The results indicated that five-ring PAHs represented 54% of total PAHs in HH-7, followed by HH-2 (52%), HH-6 (46%), HH-10 (46%), HH-1 (42%), HH-8 (41%), HH-9 (34%), HH-5 (32%), and HH-1 (25%). Four-ring PAHs also represented a relatively high ratio in indoor air of all households in decreasing order: HH-5 (28%; 17.58%), HH-7 (21.71%; 6.42%), HH-8 (20.87%; 18.33%), HH-2 (18.75%; 8.57%). Six-ring PAHs accounted for 8.8% to 14% of total PAHs concentrations in indoor air of the sampled households.

In our study, 5- and 6-ring PAHs made up relatively high fraction of total PAHs in each household. These results are in concordance with finding of Wu et al. (2015). While PAHs are distributed between vapor and particulate phases, heavier PAHs with 5–6 aromatic rings are predominantly found in particles (Lu et al., 2008).

According to literature, PAH emission from different sources can be identified based on different PAHs rings: Flu, Pyr, BaA, Cry, and DbaA are considered as tracers of coal combustion, whereas BkF, IDP, and BghiP are considered markers of vehicle exhaust (Eiguren-Fernandez et al., 2004; Ma et al., 2010; Pant et al., 2017). Considering individual characteristic PAHs, 4 rings PAHs including Flu, Pyr, BaA, and Cry were detected in all households; they were most abundant in HH-7, followed by HH-5 and HH-2, respectively. DbaA is one of the best marker, which is typically associated with coal combustion (Pant et al., 2017). DbaA was detected in all households with the exception of HH-5. The individual homologues such as lower molecular weight (LMW) Nap, Ace, and Fle are not specifically related to traffic emission. However, Nap, Ace, and Fle were detected in all households with the exception of Ace which did not occur in HH-5. In all households, the markers of vehicle emission were detected, the highest concentrations of IDP and BghiP were found in HH-7 (14.4 ng/m^3^ and 9.15 ng/m^3^), followed by HH-2 (11.8 ng/m^3^ and 7.7 ng/m^3^), HH-5 (5.4 ng/m^3^ and 4.8 ng/m^3^), and HH-10 (5.08 ng/m^3^ and 3.49 ng/m^3^).

BkF was detected in all households; high concentrations were measured in HH-7 (12.6 ng/m^3^) and HH-2 (8.9 ng/m^3^) respectively. Heavier PAH homologues (5- to 6-ring PAHs) were detected in all households, which suggested the influence of traffic exhaust (Rogula-Kozłowska et al., 2018). Our result indicated that BaP, BaA, Cry, Pyr, BkF, BbF, IDP, and BghiP were the most dominant PAHs in indoor air PM_2.5_, especially for the BaP, BaA, BkF, IDP, and BghiP, due to both sources of solid fuel combustion and traffic (Byambaa et al., 2019).

In addition to indoor and outdoor sources, many factors also influence indoor air quality such as household age, construction quality and family income, etc. (So et al., 2019). We also compared the construction age, room size of household, and people activities. Difference was found between in HH-7, HH-2, and HH-5 to other households (Table 1). The lowest total amount of PAHs (46.8 ng m^−3^) was measured in HH-3 (7 years old), while household with highest total amount of PAHs (175.7 ng m^−3^) was a relatively old building (40 year old). The concentration of 16 individual PAHs in residential air of 10 non-smokers from Chicago area homes were measured (Li et al., 2005). The lowest total concentration was found in a new building (8 years old), whereas the highest total PAHs was detected in an old one (age 80 years). Lower concentration of indoor PM_2.5_ were attributed to the presence of airtight windows in winter in Italy (Simoni et al., 2004) and large room size of household in California, USA (Klepeis et al., 2017), while higher concentration of PM-bound PAHs was associated with low-income families (Chuang et al., 1999) and movement of people (Vardoulakis et al., 2020). These results suggested that accumulated PAHs in indoor air might be related to household age and construction quality.

### Correlation factor between outdoor PM_2.5_ and indoor PM_2.5_

In this case, we used data of ambient PM_2.5_ concentrations from the National Agency of Meteorology and Environmental Monitoring. As far, there are 15 air monitoring stations in Ulaanbaatar city (http://agaar.mn/index). Nisekh and Misheel Expo air monitoring stations in Khan-Uul district, Ulaanbaatar city, are located quite close to these ten households. HH-1, HH-2, HH-3, and HH-4 are located app. 5 to 5.3 km from both air monitoring stations. The distances between HH-5, HH-6, and HH-7 and the Nisekh air monitoring station are app. 4.4 km, whereas HH-8, HH-9, and HH-10 are located quite closely to the Nisekh monitoring station (345 to 500 m).

**Fig. 5:**
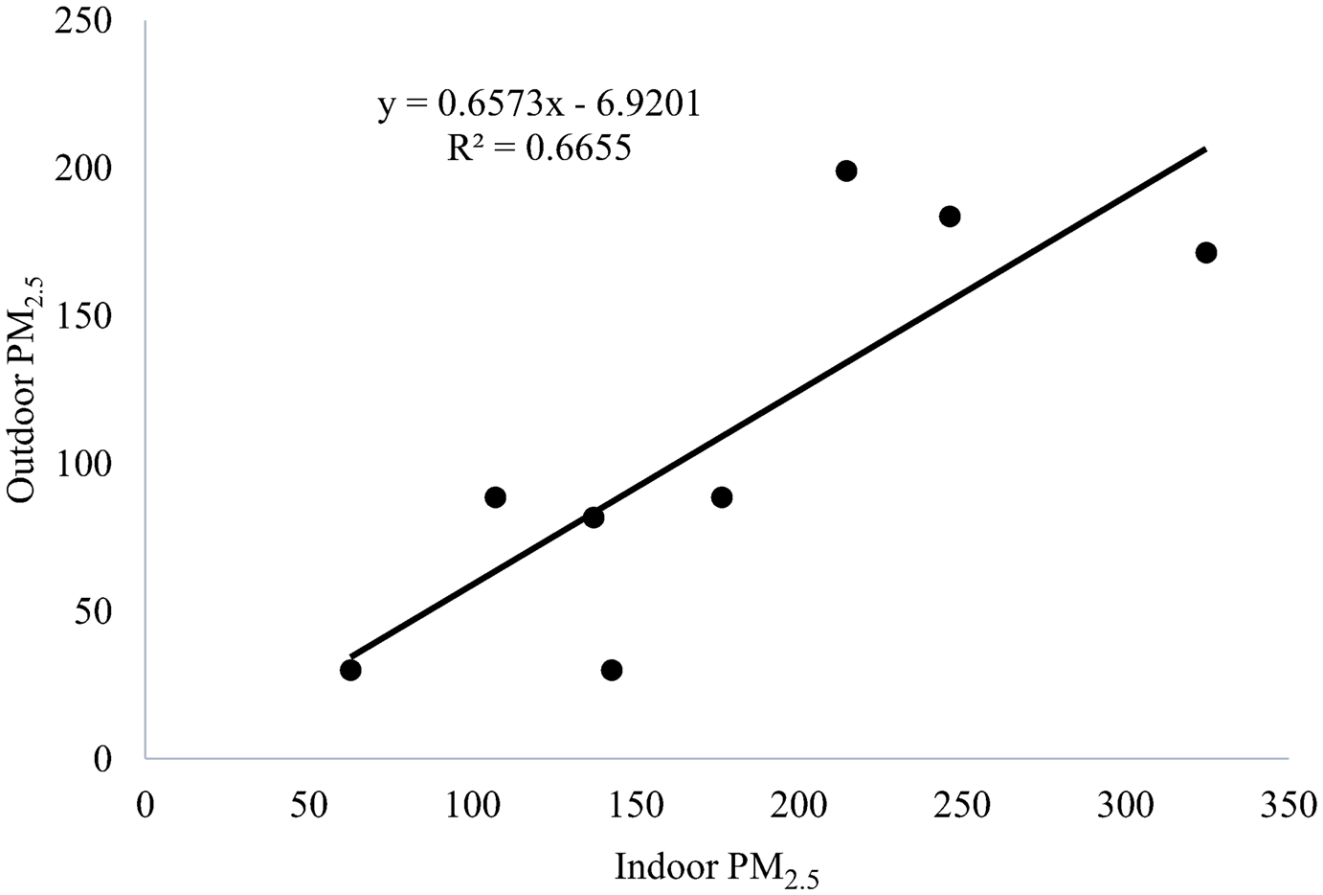
Correlation between concentrations of indoor and outdoor PM_2.5_ in the winter period

Model-based correlation between average concentrations of ambient and indoor PM_2.5_ shown in Fig. 5 was found to be moderately positive (R^2^ = 0.66, *P* value < 0.015).

In other studies, significant correlation was found between indoor and outdoor particulate matter levels in wintertime (e.g., Bai et al., 2020; Hu et al., 2018). Rogula-Kozłowska et al. (2018) showed positive correlation between PM1-bound PAH levels in Gliwice (Poland). Byambatseren et al. (2018) reported that in winter 24 h average concentration of PM_2.5_ pollution of indoor and outdoor was measured in a household which was located between ger area and the residential district in Ulaanbaatar capital at simultaneous duration and point. The result indicated that pollution of indoor ambient was strongly related to outdoor ambient. Enkhbat et al. (2016) defined outdoor pollution originating from coal burning as a main source of continuous indoor pollution. Hill et al. (2017) also states that indoor PM_2.5_ exposure is considerably influenced by infiltrated outdoor pollution.

Lim et al. (2018) determined the characteristics of indoor PM_2.5_ concentration in ger with coal stoves during winter period around the non-connected heating system area, Ulaanbaatar capital. The result showed that the average 24-h PM_2.5_ concentration was higher with improved stove than conventional stoves, and also mentioned the combustion method of the stoves. During controlled burning of Mongolian coal samples of different origin, Barabad et al. (2018) found that PM_2.5_ emission would depend on the coal used in the household.

### PAHs source identification in an indoor air of households

**Fig. 6:**
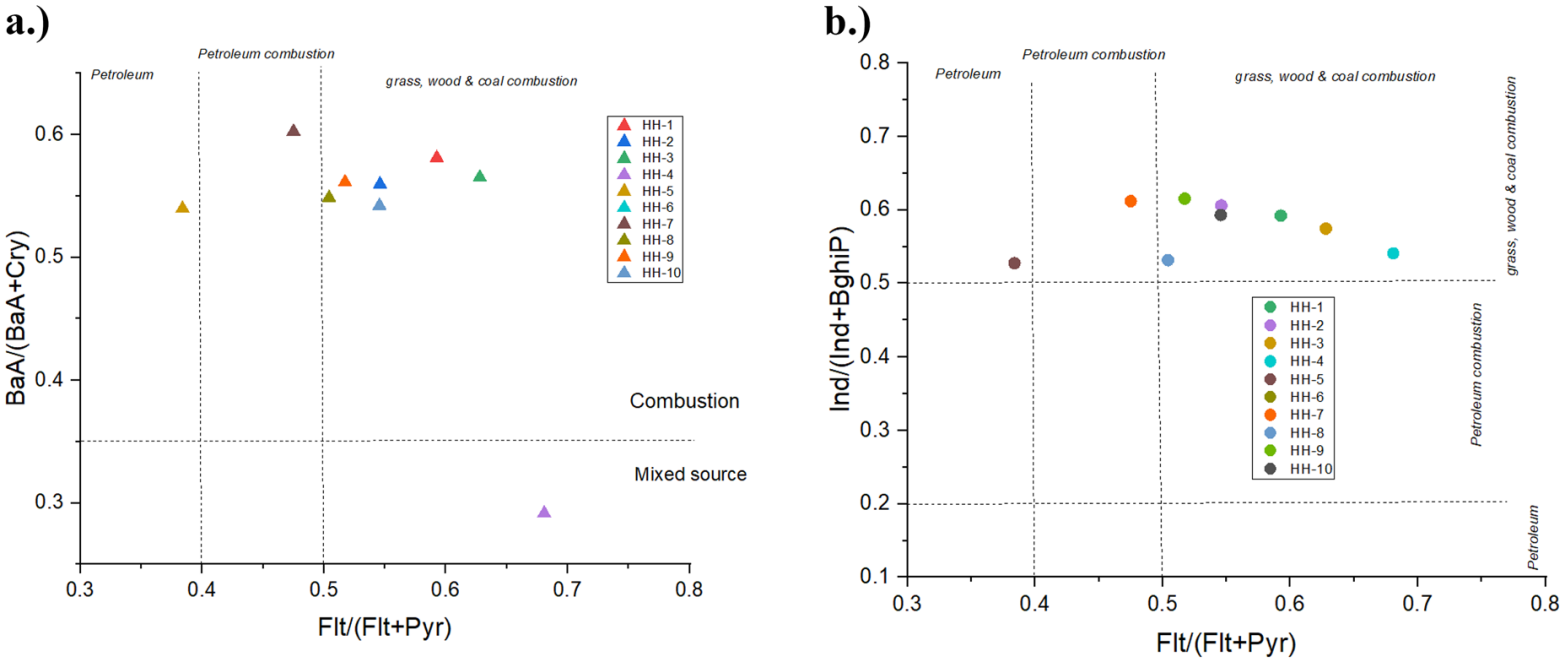
**a** Cross-plot of BaA/(BaA + Cry) ratio vs. Flt/(Flt + Pyr) in ten households. **b** Cross-plot of Ind/(Ind + BghiP) ratio vs. Flt/(Flt + Pyr) in ten household.

In order to estimate the pollution source, established ratios of PAH isomers were used (Yunker et al., 2002). Fluoranthene to (fluoranthene + pyrene) [Flt/(Flt + Pyr)] ratio below 0.4 indicates petroleum source, between 0.4 and 0.5 indicates petroleum combustion while values above 0.5 grass, wood and coal combustion. Flt/(Flt + Pyr) ratio were above 0.5 for all household which implies combustion of solid fuel (Yunker et al., 2002). Flt/(Flt + Pyr) ratio was 0.48 in HH-7 which likely implied petroleum combustion. It was very interesting to note that Flt/(Flt + Pyr) ratio was < 0.4 in case of only 1 household, HH-5 (0.38) which indicated petroleum input. The results from this study suggested that combustion of wood and coal is important source of PM_2.5_-bound PAHs in indoor air for all households with the exception of HH-7 and HH-5. Benzo{a}anthracene to ( benzo{a}anthracene + chrysene) [BaA/(BaA + Cry)] ratio over 0.35 implies combustion of vegetation and fossil fuel, less than 0.35 likely implies mixed source. This ratio value was above 0.35 for all households except only one household, HH-4, suggesting combustion input and value in HH-4 was exactly 0.29 suggesting mixed source. Figure 6 shows the crossplots of BaA/(BaA + Cry) against Flt/(Flt + Pyr), suggesting that the main important source in case of the majority of households was coal and wood combustion (Yunker et al., 2002). The crossplot of BaA/(BaA + Cry) against Flt/(Flt + Pyr) indicated that in this case considering the indoor air PM_2.5_ air samples in HH-7 and HH-5 petroleum combustion and petroleum input might be the main source. It is most interesting to note that the environment of these two households was highly differing from each other; one of the most possible sources might be the vicinity of an old mini power plant and petroleum station.

Indene[*1*,*2*,*3-cd*]pyrene to indene[*1*,*2*,*3-cd*]pyrene + benzo[*ghi*]peryene [Ind/(Ind + BghiP)] ratio above 0.5 implies combustion of grass, wood, and coal while, between 0.2 and 0.5 petroleum combustion while less than 0.2 petroleum. Figure 6 shows the cross plot for Ind/(Ind + BghiP) and Flt/(Flt + Pyr). In the majority of the households (except HH-5 and HH-7), grass, wood, and coal combustion was indicated as the major source, most possibly wood and coal for heating and cooking (Anenberg et al., 2013; Bonjour et al., 2013; Yunker et al., 2002). Several studies have reported coal combustion as the main source of air particulate matter emission in ger districts during winter season in Ulaanbaatar (e.g., Davy et al., 2011). Coal, which was burned in all households sampled in our study, has been identified as a main indoor pollution source as well. It is common that coal is used as a source of fuel for heating and cooking in winter in ger districts in Ulaanbaatar (Battsengel et al., 2021).

### Cancer and health risk assessment of PAHs contamination in indoor air of households

In order to estimate the health and cancer risk for adults and children posed by PM_2.5_-bound PAHs through inhalation in indoor environment, lifetime average daily dose (LADD) and the corresponding incremental lifetime cancer risk (ILCR) values were calculated (see Table 3). The values of LADD PAHs ranged from 3.5 × 10^−8^ to 9.0 × 10^−6^ in adults, whereas 4.4 × 10^−10^ to 1.1 × 10^−5^ in children, respectively. ILCR values associated to carcinogenic-PAHs ranged between 4.0 × 10^−10^ and 5.5 × 10^−5^ for adults, and between 4.9 × 10^−10^ and 6.8 × 10^−5^ for children.

**Table 3:** CSF adjusted of PAHs concentrations, LADD and ILCR of carcinogenic-PAHs in household indoor air (ng/m^3^) from in normal polluted area. Indicated carcinogen slope factor values ( USEPA, 1992). *nd* non-detected

PAHs	**HH-1**		**HH-2**		**HH-3**		**HH-4**		**HH-5**		**HH-6**		**HH-7**		**HH-8**		**HH-9**		**HH-10**		
	Adult	Child	Adult	Child	Adult	Child	Adult	Child	Adult	Child	Adult	Child	Adult	Child	Adult	Child	Adult	Child	Adult	Child	
Nap	1.5 × 10^−6^	1.8 × 10^−6^	1.3 × 10^−6^	1.6 × 10^−6^	1.7 × 10^−6^	2.1 × 10^−6^	1.5 × 10^−6^	1.8 × 10^−6^	1.5 × 10^−6^	1.8 × 10^−6^	1.2 × 10^−6^	1.5 × 10^−6^	1.1 × 10^−6^	1.4 × 10^−6^	1.2 × 10^−6^	1.5 × 10^−6^	1.4 × 10^−6^	1.7 × 10^−6^	1.3 × 10^−6^	1.7 × 10^−6^	
Ace	1.3 × 10^−6^	1.7 × 10^−6^	1.1 × 10^−6^	1.3 × 10^−6^	1.5 × 10^−6^	1.8 × 10^−6^	1.4 × 10^−6^	1.7 × 10^−6^	1.5 × 10^−6^	1.8 × 10^−6^	1.3 × 10^−6^	1.6 × 10^−6^	1.1 × 10^−6^	1.4 × 10^−6^	1.1 × 10^−6^	1.4 × 10^−6^	1.5 × 10^−6^	1.9 × 10^−6^	1.3 × 10^−6^	1.6 × 10^−6^	
Fle	1.3 × 10^−6^	1.6 × 10^−6^	4.6 × 10^−7^	5.7 × 10^−7^	5.7 × 10^−7^	7.1 × 10^−7^	5.9 × 10^−7^	7.3 × 10^−7^	1.5 × 10^−6^	1.8 × 10^−6^	5.2 × 10^−7^	6.5 × 10^−7^	4.5 × 10^−7^	5.6 × 10^−7^	4.4 × 10^–7^	5.5 × 10^−7^	6.4 × 10^−7^	7.9 × 10^−7^	5.4 × 10^−7^	6.7 × 10^−7^	
Phe	1.0 × 10^−7^	1.3 × 10^−7^	1.3 × 10^−7^	1.6 × 10^−7^	8.3 × 10^−8^	1.0 × 10^−7^	8.8 × 10^−8^	1.1 × 10^−7^	nd	nd	9.2 × 10^−8^	1.1 × 10^−7^	1.5 × 10^−7^	1.9 × 10^−7^	6.0 × 10^−8^	7.4 × 10^−8^	7.8 × 10^−8^	9.7 × 10^−8^	1.6 × 10^−7^	2.0 × 10^−7^	
Ant	5.8 × 10^−8^	7.1 × 10^−8^	3.5 × 10^−8^	4.4 × 10^−8^	1.4 × 10^−7^	1.710^−7^	6.6 × 10^−8^	8.1 × 10^−8^	nd	nd	5.2 × 10^−8^	6.5 × 10^−8^	4.6 × 10^−8^	5.7 × 10^−8^	3.6 × 10^−8^	4.4 × 10^−8^	5.6 × 10^−8^	6.9 × 10^−8^	5.8 × 10^−8^	7.1 × 10^−8^	
Flt	2.5 × 10^−7^	3.1 × 10^−7^	1.9 × 10^−7^	2.3 × 10^−7^	3.3 × 10^−7^	4.1 × 10^−7^	2.1 × 10^−7^	2.6 × 10^−7^	1.9 × 10^−7^	2.4 × 10^−7^	2.0 × 10^−7^	2.4 × 10^−7^	1.8 × 10^−7^	2.3 × 10^−7^	2.0 × 10^−7^	2.5 × 10^−7^	2.1 × 10^−7^	2.6 × 10^−7^	2.0 × 10^−7^	2.4 × 10^−7^	
Pyr	1.6 × 10^−6^	2.0 × 10^−6^	1.6 × 10^−6^	1.9 × 10^−6^	1.7 × 10^−6^	2.1 × 10^−6^	1.9 × 10^−6^	2.3 × 10^−6^	1.3 × 10^−6^	1.6 × 10^−6^	1.3 × 10^−6^	1.6 × 10^−6^	1.5 × 10^−6^	1.8 × 10^−6^	1.3 × 10^−6^	1.6 × 10^−6^	1.1 × 10^−6^	1.3 × 10^−6^	1.3 × 10^−6^	1.6 × 10^−6^	
BaA	5.8 × 10^−8^	7.1 × 10^−8^	9.4 × 10^−8^	1.2 × 10^−7^	5.2 × 10^−8^	6.4 × 10^−8^	6.6 × 10^−8^	8.1 × 10^−8^	1.7 × 10^−6^	2.2 × 10^−6^	7.8 × 10^−8^	9.7 × 10^−8^	9.2 × 10^−6^	1.1 × 10^−7^	9.6 × 10^−8^	1.2 × 10^−7^	4.5 × 10^−8^	5.5 × 10^−8^	8.1 × 10^−8^	1.0 × 10^−7^	
Cry	5.5 × 10^−7^	6.9 × 10^−7^	1.3 × 10^−6^	1.6 × 10^−6^	5.6 × 10^−7^	7.0 × 10^−7^	1.2 × 10^−6^	1.5 × 10^−6^	1.5 × 10^−6^	1.9 × 10^−6^	6.5 × 10^−8^	8.1 × 10^−8^	1.8 × 10^−6^	2.2 × 10^−6^	7.2 × 10^−7^	8.8 × 10^−7^	3.4 × 10^−7^	4.1 × 10^−7^	4.9 × 10^−7^	6.0 × 10^−7^	
BbF	3.8 × 10^−7^	4.7 × 10^−7^	1.0 × 10^−6^	1.3 × 10^−6^	3.3 × 10^−7^	4.1 × 10^−7^	5.8 × 10^−7^	7.2 × 10^−7^	2.4 × 10^−6^	3.0 × 10^−6^	5.9 × 10^−7^	7.3 × 10^−7^	1.9 × 10^−6^	2.4 × 10^−6^	7.0 × 10^−7^	8.7 × 10^−7^	3.1 × 10^−7^	3.9 × 10^−7^	4.0 × 10^−7^	5.0 × 10^−7^	
BkF	5.0 × 10^−7^	6.1 × 10^−7^	2.2 × 10^−6^	2.7 × 10^−6^	2.7 × 10^−7^	3.3 × 10^−7^	5.4 × 10^−7^	6.7 × 10^−7^	1.6 × 10^−6^	2.0 × 10^−6^	1.0 × 10^−6^	1.3 × 10^−6^	3.3 × 10^−6^	4.0 × 10^−6^	9.4 × 10^−7^	1.2 × 10^−6^	3.6 × 10^−7^	4.4 × 10^−7^	6.7 × 10^−7^	8.3 × 10^−7^	
BeP	3.6 × 10^−7^	4.4 × 10^−7^	1.7 × 10^−6^	2.1 × 10^−6^	2.1 × 10^−7^	2.6 × 10^−7^	1.3 × 10^−6^	1.6 × 10^−6^	1.4 × 10^−6^	1.7 × 10^−6^	7.8 × 10^−7^	9.7 × 10^−7^	2.2 × 10^−6^	2.7 × 10^−6^	7.8 × 10^−7^	9.6 × 10^−7^	2.8 × 10^−7^	3.5 × 10^−7^	5.7 × 10^−7^	7.0 × 10^−7^	
BaP	2.8 × 10^−6^	3.4 × 10^−6^	6.9 × 10^−6^	8.5 × 10^−6^	1.1 × 10^−6^	1.3 × 10^−6^	3.1 × 10^−6^	3.8 × 10^−6^	3.1 × 10^−6^	3.9 × 10^−6^	3.1 × 10^−6^	3.9 × 10^−6^	9.0 × 10^−6^	1.1 × 10^−−5^	2.7 × 10^−6^	3.4 × 10^−6^	1.3 × 10^−6^	1.6 × 10^−6^	2.8 × 10^−6^	3.5 × 10^−6^	
DBahA	8.1 × 10^−7^	1.0 × 10^−6^	2.1 × 10^−6^	2.6 × 10^−6^	2.5 × 10^−7^	3.1 × 10^−7^	5.8 × 10^−7^	7.2 × 10^−7^	9.0 × 10^−7^	1.1 × 10^−6^	9.4 × 10^−7^	1.2 × 10^−6^	3.0 × 10^−6^	3.7 × 10^−6^	6.8 × 10^−7^	8.4 × 10^−7^	5.4 × 10^−7^	6.6 × 10^−7^	8.7 × 10^−7^	1.1 × 10^−6^	
IDP	1.3 × 10^−6^	1.6 × 10^−6^	2.9 × 10^−6^	3.6 × 10^−6^	4.4 × 10^−7^	5.4 × 10^−7^	8.1 × 10^−7^	1.0 × 10^−6^	1.8 × 10^−6^	2.3 × 10^−6^	1.4 × 10^−6^	1.7 × 10^−6^	4.2 × 10^−6^	5.2 × × 10^–6^	1.3 × 10^−6^	1.6 × 10^−6^	6.5 × 10^−7^	8.0 × 10^−7^	1.6 × 10^−6^	2.0 × 10^−6^	
BghiP	1.9 × 10^−6^	2.3 × 10^−6^	4.9 × 10^−6^	6.0 × 10^−6^	9.7 × 10^−7^	1.2 × 10^−6^	1.6 × 10^−6^	2.0 × 10^−6^	2.3 × 10^−6^	2.8 × 10^−6^	2.0 × 10^−6^	2.5 × 10^−6^	6.0 × 10^−6^	7.4 × 10^−6^	1.3 × 10^−6^	1.6 × 10^−6^	1.1 × 10^−6^	1.4 × 10^−6^	2.1 × 10^−6^	2.6 × 10^−6^	

	**Incremental lifetime cancer risk (ILCR)**																				**CSF**
BaA	3.5 × 10^−8^	4.4 × 10^−8^	5.7 × 10^−8^	7.1 × 10^−8^	3.2 × 10^−8^	3.9 × 10^−8^	4.0 × 10^−8^	5.0 × 10^−8^	1.1 × 10^−6^	1.3 × 10^−6^	4.8 × 10^−8^	5.9 × 10^−8^	5.6 × 10^−8^	7.0 × 10^−8^	5.8 × 10^−8^	7.2 × 10^−8^	2.7 × 10^−8^	3.4 × 10^−8^	4.9 × 10^−8^	6.1 × 10^−8^	0.61
Cry	3.4 × 10^−9^	4.2 × 10^−9^	7.7 × 10^−9^	9.5 × 10^−9^	3.4 × 10^−9^	4.2 × 10^−9^	7.6 × 10^−9^	9.4 × 10^−9^	9.2 × 10^–9^	1.1 × 10^−8^	4.0 × 10^−10^	4.9 × 10^−10^	1.1 × 10^−8^	1.3 × 10^−8^	4.4 × 10^−9^	5.4 × 10^−9^	2.0 × 10^−9^	2.5 × 10^−9^	3.0 × 10^−9^	3.7 × 10^−9^	0.0061
BbF + BkF	4.1 × 10^−7^	5.1 × 10^−7^	1.2 × 10^−6^	1.5 × 10^−6^	3.5 × 10^−7^	4.3 × 10^−7^	6.1 × 10^−7^	7.6 × 10^−7^	2.5 × 10^−6^	3.1 × 10^−6^	6.5 × 10^−7^	7.3 × 10^−7^	2.1 × 10^−6^	2.4 × 10^−6^	7.6 × 10^−7^	8.8 × 10^−7^	3.3 × 10^−7^	3.9 × 10^−7^	4.4 × 10^−7^	5.0 × 10^−7^	0.06
BaP	**1.7** × **10**^−**5**^	**2.1** × **10**^−**5**^	**4.2** × **10**^−**5**^	**5.2** × **10**^−**5**^	6.5 × 10^−6^	8.0 × 10^−6^	**1.9** × **10**^−**5**^	**2.3** × **10**^−**5**^	**1.9** × **10**^−**5**^	**2.4** × **10**^−**5**^	**1.9** × **10**^−**5**^	**2.4** × **10**^−**5**^	**5.5** × **10**^−**5**^	**6.8** × **10**^−**5**^	**1.7** × **10**^−**5**^	**2.1** × **10**^−**5**^	8.1 × 10^−6^	**1.0** × **10**^−**5**^	**1.7** × **10**^−**5**^	**2.1** × **10**^−**5**^	**6.1**
DBahA	4.9 × 10^−6^	6.1 × 10^−6^	**1.3** × **10**^−**5**^	**1.6** × **10**^−**5**^	1.5 × 10^−6^	1.9 × 10^−6^	3.6 × 10^−6^	4.4 × 10^−6^	5.5 × 10^−6^	6.8 × 10^−6^	5.7 × 10^−6^	7.1 × 10^−6^	**1.8** × **10**^−**5**^	**2.3** × **10**^−**5**^	4.2 × 10^−6^	5.1 × 10^−6^	3.3 × 10^−6^	4.0 × 10^−6^	5.3 × 10^−6^	6.5 × 10^−6^	6.1
IDP	7.8 × 10^−7^	9.6 × 10^−7^	1.8 × 10^−6^	2.2 × 10^−6^	2.7 × 10^−7^	3.3 × 10^−7^	5.0 × 10^−7^	6.1 × 10^−7^	1.1 × 10^−6^	1.4 × 10^−6^	8.6 × 10^−7^	1.1 × 10^−6^	2.6 × 10^−6^	3.2 × 10^−6^	7.8 × 10^−7^	9.6 × 10^−7^	4.0 × 10^−7^	4.9 × 10^−7^	9.7 × 10^−7^	1.2 × 10^−6^	0.61

Lifetime-average daily dose for children exceeded the health based guideline level (1.0 × 10^−5^) defined by WHO (Boström et al., 2002) in only one household (see Table 3), whereas LADD for adults and children of other households were within acceptable limit. The cancer risks from the exposure of children to air pollutants in all households except HH-3 were found high. It should be noted, however, that USEPA (2005) suggests the use of adjustment factors due to toxicokinetic and toxicodynamic differences between children and adults.

This means that the excess lifetime cancer risks were one order of magnitude higher than 10^−6^ which was set by US EPA as a risk level for carcinogenic individual compounds. High level of these pollutants was detected in indoor air, which might cause specific long-term health effects, e.g., lung cancer. The lifetime inhalation cancer risk was estimated in the ger area due to winter pollution in the study of Byambaa et al. (2019). Estimated values were as follows: 1.2 × 10^−5^ for child and 2.1 × 10^−5^ for adult exposures.

Considering cancer risk, our results were comparable to, e.g., the study conducted in residential homes in winter and summer period in Shimizu, Japan, an industrial area (Ohura et al., 2004). However, Lu et al. (2008) reported two order of magnitude higher values for PAHs exposure in indoor air of public places in Hangzhou (ranging from 0.6 × 10^−3^ to 2.4 × 10^−3^).

Indoor air exposure to PAHs in all studied households seems to raise health issues. BaP equivalent concentrations are 1.2, 0.12, and 0.012 ng m^−3^ producing an excess life time cancer risk 1:10,000; 1:100,000; and 1:1,000,000; respectively (Bari et al., 2010).

**Fig. 7:**
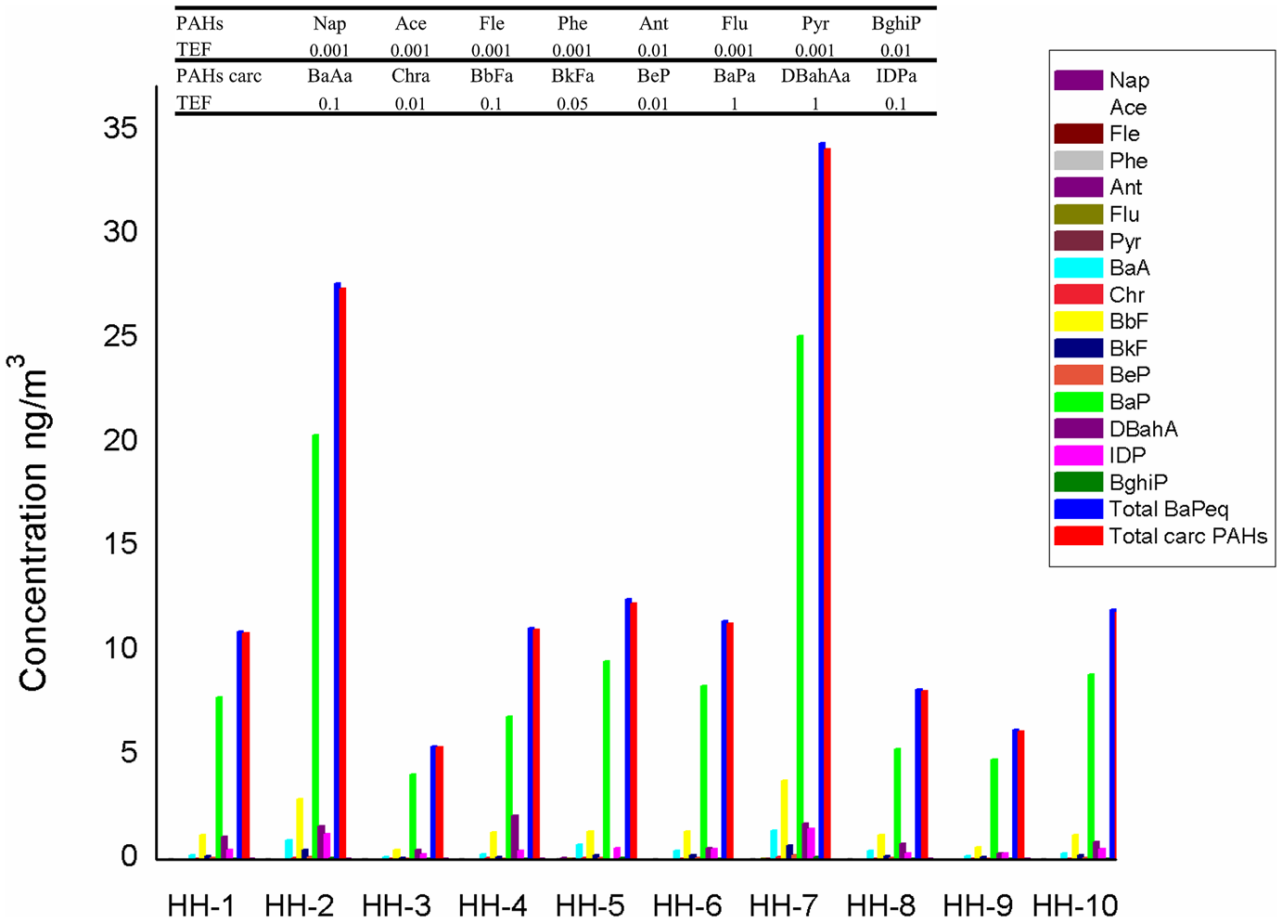
Toxic equivalence factor (TEF) and calculated BaP equivalent (BaPeq) exposure value *for the household indoor PM*_*2.5*_* air*

In our study, ∑BaPeq in indoor air of households ranged between 5.4 and 34 ng m^−3^. Highest ∑BaPeq were found in HH-7 (34.41 ng m^−3^), HH-2 (27.64 ng m^−3^), and HH-5 (12.51 ng m^−3^), while lowest concentrations in HH-3 (5.41 ng/m^3^) and HH-9 (6.21 ng m^−3^) (Fig. 7). BaP concentration alone of total ∑BaPeq in all household indoor air exceeded 1 ng m^−3^ which is prescribed by the Chinese Air Quality Standards (Wu et al., 2015). The highest concentrations in indoor air were found in HH-7 (25.13 ng m^−3^) and HH-2 (20.37 ng m^−3^), while lowest concentrations occurred in HH-3 (4.06 ng m^−3^) and HH-9 (4.78 ng m^−3^) respectively.

BaP concentration alone in PM_2.5_ fraction of indoor air of the households ranged between 4.06 and 25.13 ng m^−3^. Similar result was found in the study of Yury et al. (2018) in which mean ∑BaPeq concentration in PM_2.5_ sampled for 24 h in an empty room ranged from 5.5 to 25.4 ng m^−3^. It should be noted, however, that exceptionally high level of BaP (3249 ng m^−3^) was measured in rural households in Xuanwei (Mumford et al., 1990).

Risk of lifetime cancer to children and adult was substantially higher in cold period and that indoor air quality are more pay attention to effectively mitigate the health risk to prevent early exposure as follows: most important few approaches might be used (i) to replace raw coal with processed coal (Byambajav et al., 2021), (ii) more focus on electricity usage (Amod et al., 2015), and (iii) can be used the different types of air filter to reduce indoor PM_2.5_ (Prabjit et al., 2018; Ching-Huang et al., 2021).

### Ecotoxicity assessment of indoor PM_2.5_ of households

**Fig. 8:**
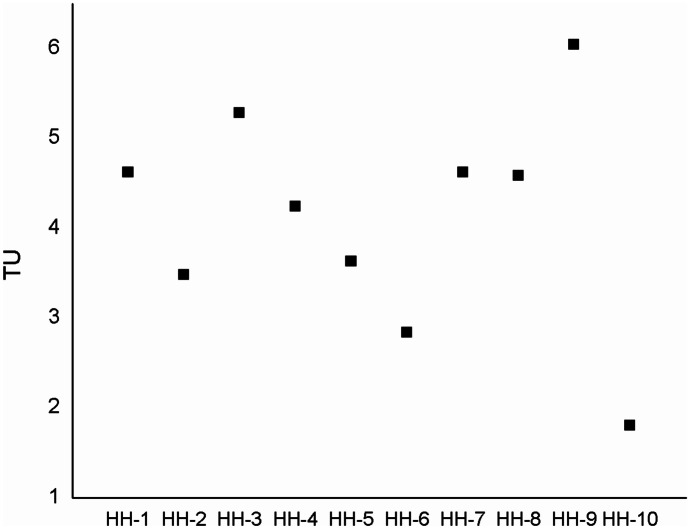
The toxic unit values of an indoor PM_2.5_ from ten household

The present study was the first application of the *V. fischeri* bioluminescence inhibition assay on indoor samples in Mongolia using the bioluminescence inhibition bacterium test. Based on calculated toxic units (TUs) (Chang et al., 2013), all samples were classified as toxic (Fig. 8). Differences in the ecotoxicity can be partially explained by the PAH concentrations: percentage of HMW PAHs (5- to 6-rings) was the highest in HH-2 (66.5%), TU was also outstanding in this sample (5.4). Ratio of HMW PAHs was also high in HH-6 (57%), TU was 5.5. In HH-5, TU was 5.5, whereas HMW PAHs amounted to 42.2%.

In the study of Evagelopoulos et al. (2009), good correlation was found between PAHs content and ecotoxicity for urban samples collected in Kozani (Greece). Alves et al. (2021) assessed the ecotoxicity of indoor PM_10_ samples collected during cooking in domestic kitchen using the bioluminescence inhibition bioassay. The result proved that LMW PAHs did not show any correlation with toxicity values, whereas good correlation was found between HMW PAHs and toxicity values (r^2^ = 0.94). Kováts et al. (2020) used the *V*. *fischeri* bioassay to evaluate the seasonal differences in rural particular matter ecotoxicity. The results revealed that PAHs content (5- to 6- rings PAHs) was higher in winter and autumn, *Vibrio* results also showed higher ecotoxicity for these seasons. A similar tendency appeared in other studies (Isidori et al., 2003; Triolo et al., 2008).

**Fig. 9:**
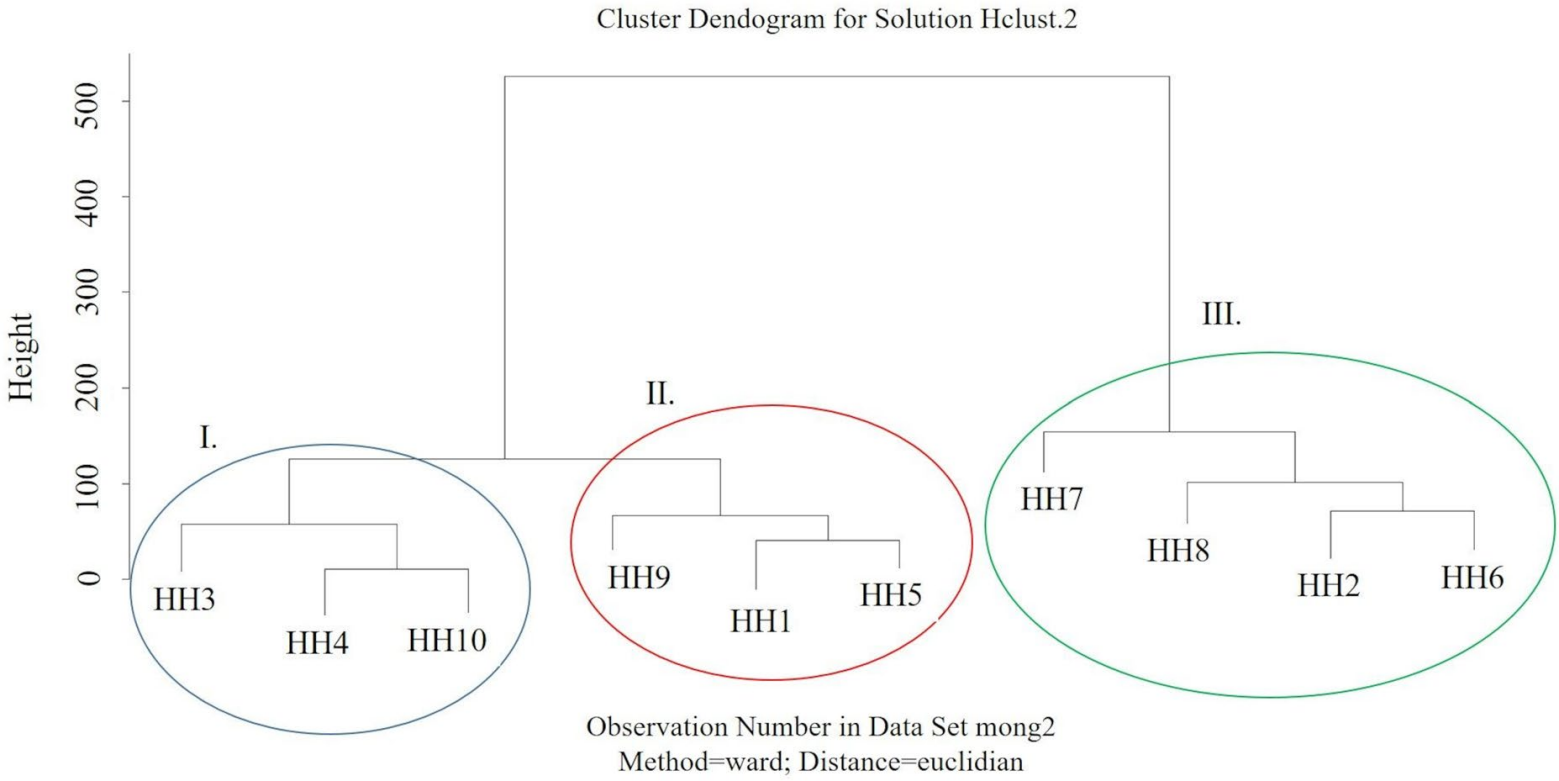
Dendogram of different households based on the individual PAH concentrations, TU, and indoor PM_2.5_ concentrations

In our study, good correlation was found between indoor PM_2.5_ levels and TU values (*t* = 2.4803, df = 8, *p* value = 0.03809; R^2^ = 0.6593202). *Vibrio* inhibition seems to reflect the overall ecotoxicity of the samples, which in addition to PAHs, might be attributed to heavy metals such as Cr, Cu, Zn, Ni, Cd, and Pb (Wang et al., 2021). Figure 9 shows the output of cluster analysis of different households based on the individual PAH concentrations, TU, and indoor PM_2.5_ concentrations. Three groups could be identified. Significant difference was found between group I. and group III (*t* =  − 2.2638, df = 21, *p* = 0.0343) and non-significant differences were found between group I and group II (*t* =  − 1.2537, df = 21, *p* = 0.2237) and group II and group III (*t* =  − 0.70335, df = 21, *p* = 0.4896). The proximity of HH2, HH6, and HH8 also reflects the similarities between households having high indoor toxicity.

## Conclusions

Concentrations of 19 individual PAHs in indoor PM_2.5_ samples of ten households were determined in Khan-Uul district, Ulaanbaatar. Sampling was carried out in winter of 2018. Typical fuel for heating in all households is coal. The results show that HMW PAHs (5- and 6-ring) contributed to a large fraction of total PAHs in each sample, and the potentially most carcinogenic PAH, BaP was predominant among the 5-ring PAHs. Significant correlation was found between indoor and ambient particulate matter levels in wintertime. Much more of the daily time spent by people in developed and developing countries is in enclosed buildings and by the very young and elderly, even more. Thus, indoor air quality in an enclosed building is of significance to human health.

Health risk of children attributed to PAHs inhalation was assessed by taking into account the lifetime-average daily dose (LADD) and corresponding lifetime cancer risk. LADD for children in indoor air of only one household were slightly higher than health-based guideline level (1.0 × 10^−5^) set by the WHO. The cancer risks from the exposure of children to air pollutants in all households except HH-3 were found high. In the *Vibrio fischeri* bioluminescence inhibition assay, according to the TU values of indoor PM_2.5_ from ten households of Mongolia, all samples were classified as toxic. It should be noted that as the Vibrio test measures the aggregate toxicity of the samples, strong relationship could be detected between TUs and PM_2.5_ concentrations.

Children spend a significant part of their time in enclosed buildings such as home, school etc., also, they are more sensitive to air pollution compared to adults. To our best knowledge, this is the first study dealing with indoor air quality of Ulaanbaatar city which is among the most polluted capitals in the world. Our results are partially comparable to other studies completed in other cold regions, showing elevated risk to inhabitants. The results of the current study will most possibly provide a starting point for future air quality studies and for implementing a strategy to control air quality in places where children reside.

## Data Availability

Data generated during the study are included in the manuscript.

## Abbreviations

Acl: Acenaphthylene
Ace: Acenaphthene
Ant: Anthracene
AT: Average timing
BaA: Benz[a]anthracene
BbF: Benzo[b]fluoranthene
BkF: Benzo[k]fluoranthene
BeP: Benzo[e]pyrene
BaP: Benzo[a]pyrene
BghiP: Benzo[g,h,i]perylene
BW: Body weight
CF: Unit of conversion factor
CSF: Cancer slope factor
GC_MS: Gas chromatography-mass spectrometry
Cry: Chrysene
DBahA: Dibenzo[a,h]anthracene
ED: Exposure duration
EF: Exposure frequency
EPA: Environmental Protection Agency
ET: Exposure time
Fle: Fluorene
Flu: Fluoranthene
HH: Household
HMW: High molecular weight
ILCR: Incremental life cancer risk
IR: Intake rate
IDP: Indeno[1,2,3CD]pyrene
LADD: Lifetime average daily dose
LMW: Low molecular weight
NSFB: Non-solid fuel burning
Nap: Naphthalene
Methy-Nap: 2-Methyl Naphthalene
Me-Nap: 1-Methyl Naphthalene
QA: Quality assurance
QC: Quality control
PAH: Polycyclic aromatic hydrocarbon
PM: Particulate matter
Phe: Phenanthrene
Pyr: Pyrene
SFB: Solid fuel burning
TEF: Toxic equivalency factor
TU: Toxic unit
WHO: World Health Organization
WAQ: World Air Quality
